# Evaluation of Biochemical Juice Attributes and Color-Related Traits in Muscadine Grape Population

**DOI:** 10.3390/foods10051101

**Published:** 2021-05-16

**Authors:** Jiovan Campbell, Ali Sarkhosh, Fariborz Habibi, Pranavkumar Gajjar, Ahmed Ismail, Violeta Tsolova, Islam El-Sharkawy

**Affiliations:** 1 Center for Viticulture and Small Fruit Research, College of Agriculture and Food Sciences, Florida A&M University, Tallahassee, FL 32308, USA; jiovan1.campbell@famu.edu (J.C.); pranavkumar1.gajjar@famu.edu (P.G.); ahmed.ismail@agr.dmu.edu.eg (A.I.); violeta.tsolova@famu.edu (V.T.); 2 Horticultural Sciences Department, University of Florida, Gainesville, FL 32611, USA; sarkhosha@ufl.edu; 3 Department of Horticultural Science, School of Agriculture, Shiraz University, Shiraz 71441-65186, Iran; fariborz_h659@yahoo.com; 4 Department of Horticulture, Faculty of Agriculture, Damanhour University, Damanhour, Behera 22516, Egypt

**Keywords:** acidity, anthocyanin, chroma index, hue angle, total soluble solids

## Abstract

Biochemical juice attributes and color-related traits of muscadine grape genotypes have been investigated. For this study, 90 muscadine genotypes, including 21 standard cultivars, 60 breeding lines, and 9 *Vitis x Muscadinia* hybrids (VM), were evaluated. The biochemical properties of total soluble solids (TSS), titratable acidity, and TSS/Acid (T/A) ratio showed modest diversity among genotypes with a range of 10.3 °Brix, 2.1 mg tartaric acid/L, and 4.6, respectively. Nonetheless, the pH trait exhibited a tight range of 0.74 among the population with a minimum and maximum pH of 3.11 ± 0.12 and 3.85 ± 0.12. Color-related traits showed more deviation between individuals. Total anthocyanin content (TAC), luminosity index (L*), hue angle (*h*°), and chroma index (C*) displayed a range of 398 µg/g DW, 33.2, 352.1, and 24, respectively. The hierarchical clustering map classified the population into two large groups of colored and non-colored grapes based on L* and *h*°, suggesting the predominance of these two characters among the population. The colored berries genotypes clade was further divided into several sub-clades depending on C*, TAC, and TSS levels. The principal component analysis (PCA) separated the four-color characteristics into two groups with a negative correlation between them, L* and C* versus TAC and *h*°. Further, PCA suggested the positive influence of acidity in enhancing the different nutraceutical components. Despite the nature of anthocyanins as a member of phenolic compounds, a lack of significant correlation between TAC and nutraceutical-related traits was detected. The dissimilatory matrix analysis highlighted the muscadine individuals C11-2-2, E16-9-1, O21-13-1, and Noble as particular genotypes among the population due to enhanced color characteristics.

## 1. Introduction

Muscadines (*Muscadinia rotundifolia* Michx) are Native American grapes that have been cultivated for over 400 years [1,2]. They are commonly grown in the southeastern region of the United States due to their high adaptability to diverse biotic/abiotic stresses and pleasant fruit/vinification qualities. Muscadine grapes are used for fresh consumption and processed into juice, wine, or jam. Reportedly, there are almost 100 improved cultivars and approximately 5000 acres of muscadine grapes in commercial production in the United States [3]. The recent recognition that muscadine grapes are important sources of beneficial health-promoting phytochemicals has considerably increased their demand by consumers as a favorite healthy food [4,5,6]. Their unique blend of bioactive elements is effective for preventing several chronic diseases [7,8,9].

Biochemical attributes, including flavor, taste composition, and skin color are the most dominant quality parameters of grapes for market and to attract consumers. Among the flavor characteristics, components such as metabolites, aroma, total soluble solids, acidity, and anthocyanins are the ordinary parameters that largely contribute to grape and wine taste [10]. During development, fruit grows in a coordinated program of physiological, biochemical, and structural systems to optimize changes in size, shape, color, texture, and metabolic dynamics of grape berries. Despite the importance of grape nutraceutical value, the factors of TSS, acid, TSS/acid ratio, and skin color are still the main quality attributes in grapes that determine consumer demand [11]. Berry chemical composition at harvest could be affected by several factors such as growing season, maturity stage, genotypical variation, and other factors like storage conditions [12,13]. In ripe grapes, acids are present in traces relative to sugars, but they considerably contribute to the overall taste [14]. Muscadine growers typically use TSS (sweetness) of juice as a sign of maturity. For instance, berries exhibit TSS levels between 15–18 °Brix are considered mature and ready for harvest [15]. Sugar content in grape berries is the crucial aspect determining their quality [16]. In muscadines, glucose, fructose, and sucrose constituted approximately 90% of the total sugars in berries with trace quantities of galactose and maltose [10,17].

The attractive color is a primary sensory characteristic of fruit products [18]. Muscadine berries typically display a dark-purple/black color or bronze color. Marketplaces typically sell both colors, and many consumers prefer one to the other. Despite the predominance of the bronze and black colors, other colors are available, varying from lavender to purple and pink through red shades. Breeding cultivars with new skin colors may open up new markets for muscadines and prioritize breeding programs [19]. Berry color is a quality factor of a primary effect, since sight is the first of the senses to be used, and visual appreciation is pivotal in the choice [11]. In addition, berry color is the visual manifestation of organic compounds known as anthocyanins, mainly accumulated within the skin and occasionally in the flesh. Anthocyanins are colored water-soluble pigments belonging to the phenolic group. The pigments are in glycosylated forms. Various red and black grapes contain anthocyanins, whereas white grape varieties do not accumulate pigment [20]. Among the different anthocyanin pigments, cyanidin-3-glucoside is the major anthocyanin detected in most plants. The colored anthocyanin pigments have been traditionally used as a natural food colorant. The color and stability of these pigments are influenced by pH, light, temperature, and structure [21]. Anthocyanins occur in both the skin and the seed, but skin has a much higher content than the seed [5]. In grapes, *O*-glycosylation occurs for anthocyanins, and the sugar moiety is typically glucose. The color of the grape skin in red, purple, and black is attributed to different types of anthocyanins, including delphinidin, cyanidin, petunidin, peonidin, and malvidin [4]. The tonality and intensity of the color in the juice can provide information about the quality of the raw material used in its preparation. Muscadines’ juice color quality and stability are affected by the total amount of anthocyanins in the berry, the relative proportion of the individual anthocyanins, and the lack of intramolecular co-pigmentation [21,22,23,24,25,26]. Ballinger et al. [27] examined the anthocyanin profile of 39 *M. rotundifolia* clones and noticed that delphinidin was the predominant anthocyanin in over 90% of the samples. According to Hoffman et al. [28], fresh market muscadines differ considerably from those grown for processing. Fresh market muscadine berries need to be large (over 6 g per berry or <50 berries per quart), have a dry stem scar or a wet scar that will dry quickly, and have a storage life greater than 14 days. Size, color uniformity, and lack of cosmetic defects and blemishes are essential in fresh market cultivar selection.

A comparative study on juice attributes and skin color of muscadine grapes can provide valuable information for market demand and muscadine breeding program. Therefore, the objective of this study was to determine and compare biochemical juice attributes and color-related traits of 90 muscadine genotypes grown in Florida. This research will make substantial fundamental knowledge that will express muscadine grape characteristics and create a classification about quality attributes to exploit flavor and taste for the viticulture industry.

## 2. Materials and Methods

### 2.1. Plant Material

The muscadine population used in this study was generated as part of the grape breeding program at the Florida Agricultural and Mechanical University (FAMU), Center for Viticulture and Small Fruit Research (CVSFR). For this study, 90 muscadine genotypes, including 21 standard cultivars, 60 breeding lines, and 9 *Vitis x Muscadinia* hybrids (VM hybrids), were selected based on vines age with at least 5-year-old vines to ensure stable productivity. A complete list of used genotypes and parental pedigree is presented (Table S1). The muscadine grape berries from each genotype were collected at the time of optimum harvest maturity, as determined by berry softness and color. All evaluated parameters were measured for three consecutive years (2017–2019). For chemical properties of fruit such as TSS, titratable acidity, T/A ratio, and pH were evaluated, using 50 berries/replicate (three independent biological replicates/genotype).

### 2.2. Biochemical-Related Traits

The biochemical-related traits were measured using berry juice and expressed using a set of sub-traits, including measurable and calculated traits. The measurable traits comprise total soluble solids—TSS (°Brix), acidity—Acid (mg tartaric acid/L), and pH. The calculated trait covers the TSS/Acid ratio-T/A ratio. A representative of 50 berries/genotype were randomly selected from the harvested lot at commercial maturity. The berries were then introduced to an Omega Fruit and Vegetable Juicer J4000 High-Speed Pulp Ejection Juicer (Omega Products International, Corona, CA, USA), allowing the separation of pomace and juice. The juice was used for the following biochemical measurements.

TSS was measured using HI96801 portable digital refractometer (Hanna Instruments, Woonsocket, RI, USA). The refractometer unit was zeroed before using by adding 1–2 drops of distilled deionized water to the optical location. Then, 1–2 drops of berry sample juice were added to measure the TSS value. The optical location was cleaned and zeroed with distilled deionized water between measurements. All TSS measurements were generated from three biological replicates, and each was run in three technical replicates.

Acidity concentration was assessed according to the method described by Chito et al. [29], using Tartaric Acid Assay Kit (Megazyme, Chicago, IL, USA) with minor modification to accommodate the reaction in 96-well microplates (Genesee Scientific, San Diego, CA, USA). Acidity assay was performed using 10-μL of grape juice sample in 250-μL final reaction volume. The reaction composition and steps were as follows: 175-μL of distilled water, 40-μL of Reagent 1 (Indicator solution), and 10-μL of juice sample were added to each well. The reactions were mixed by slow shaking and incubated in the dark for 1 min at room temperature. Then, 25-μL of Reagent 2 (0.1 N of NaOH) was added to each reaction, followed by incubation in the dark for 4 min at room temperature. The same procedure was followed for the blank and standard reactions; however, the 10-μL of juice sample was replaced by distilled water and standard solution. All acidity assays were generated from three biological replicates, and each was run in three technical replicates. The measure of the absorbance was performed at λ = 505 nm using ACCURIS SMART Plate Reader spectrophotometer (Thomas Scientific, Swedesboro, NJ, USA).

The T/A ratio was calculated by dividing the values of TSS and acidity. The juice pH was measured using Thermo Scientific Orion Star A111 Benchtop pH Meter (Thermo Fisher Scientific, Waltham, MA, USA). A volume of 20 mL juice was placed in a beaker with a magnetic stir bar for stirring and getting a homogenized juice. Then, the pH probe was immersed in the juice. The pH value was recorded when the ready indicator alert was stable. The pH probe was rinsed between measurements with triple distilled deionized water, dried, and used for the following sample. The pH meter was calibrated using 4, 7, and 10 pH standards for each time before using to avoid errors. All pH measurements were generated from three biological replicates, and each was run in three technical replicates.

### 2.3. Color-Related Traits

#### 2.3.1. Color Parameter Values

The skin color was measured at four different positions around the equator of the berries. Color coordinates (L*, a*, and b*) were determined by a Konica Minolta, CR-10 Plus fruit colorimeter (Konica Minolta, Inc. Chiyoda City, Tokyo, Japan) used for a deeper unbiased color index. The instrument was calibrated with a white blank calibration tile before each measurement. Luminance coordinate L* means the lightness from 0 (black) to 100 (white). The chromaticity value a* means red when positive and green when negative. The chromaticity coordinate b* means yellow when positive and blue when negative [30]. The chroma (C*) and hue (*h*°) values were calculated from a* and b* values, with C* calculated as (a^2^ + b^2^)^1/2^. For the *h*° angle, the calculation depended on the obtained charge of a* and b* values. If the a* and b* values were positive, the *h*° = Arc Tan (b/a). If the a* value was positive and the b* value was negative, the *h*° = 360 + Arc Tan (b/a). However, if the a* value was negative and the b* was positive, or both values were negative, the *h*° = 180 +Arc Tan (b/a). All color parameters were generated from five independent berries. For each berry, the skin color was measured at four different positions around the equator of the berries.

#### 2.3.2. Estimation of Total Anthocyanins Content (TAC)

Total anthocyanin content (TAC) was assessed according to the method described by Giusti and Wrolstad [31], using Anthocyanins Assay Kit (Cosmo Bio, Carlsbad, CA, USA) with minor modification to accommodate the reaction in 96-well microplates (Genesee Scientific, San Diego, CA, USA). Muscadine methanolic berry extracts were prepared as described previously [32]. Dried extracts (10 mg) dissolved in 1 mL DMSO (Dimethyl sulfoxide) were used to quantify the TAC assay. Anthocyanin assay was performed using 20-μL of 10 mg/mL extract solution in a 220-μL final volume. The reaction composition and steps were as follows: in two side-by-side wells, a volume of 200-μL of Reagent A (KCl—25 mM) and 200-μL of Reagent B (Na Acetate—0.4 M, pH 4.5) were added. Then, a 20-μL of extract solution was added to each well. The reactions were mixed by slow shaking for 1 min and incubated in the dark for 10 min at room temperature. The absorbance measurements were performed at λ = 510 nm (maximum anthocyanin absorption) and λ = 700 nm (for turbidity correction) using ACCURIS SMART Plate Reader spectrophotometer (Thomas Scientific, Swedesboro, NJ, USA). TAC estimation was generated from three biological replicates, and each sample was run in three technical replicates and expressed as a microgram of delphinidin equivalents per gram dry weight (µg/g DW).

### 2.4. Statistical Analysis

Data of several evaluated traits were collected and analyzed to test the genotype effect using repeated measures of analysis of variance (ANOVA) in SAS (SAS version 9.4, SAS Institute Inc., Raleigh, NC, USA) using PROC GLIMMIX. Data analysis were conducted based on the average of the three years’ results. Means for the analyses was determined using the LSMEANS statement and means separation performed using the Tukey–Kramer adjusted multiple means comparison test. All data presented as the mean ± SD of three growing seasons (2017–2019). A web-based program called Heatmapper (www.heatmapper.ca/pairwise/, accessed on 17 April 2021) was used to produce the graphic representation of the dissimilarity matrix. Principal component analysis (PCA) and hierarchical clustering were carried out using XLSTAT software to examine the grouping of genotypes. PCA was run on the log2-transformed area using the individual variables. Hierarchical clustering was run using the complete linkage method with correlation.

## 3. Results

### 3.1. Biochemical-Related Traits

#### 3.1.1. Total Soluble Solids (TSS) Trait

**Figure 1:**
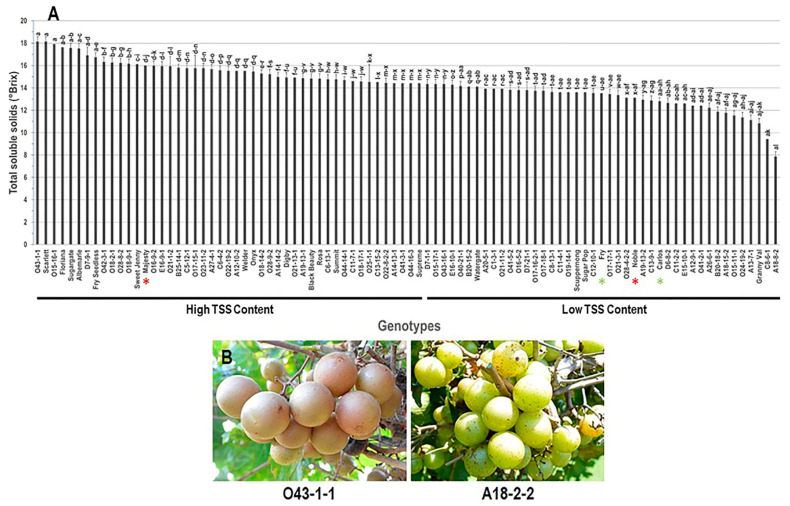
(**A**) Characterization of total soluble solids (TSS) trait among muscadine population (*n* = 90). The bars represent the mean (±SD) TSS levels resulted from three biological and three technical replicates among three years (*n* = 27). The *y*-axis refers to the total soluble solids (°Brix). The *x*-axis refers to muscadine genotypes. Means within columns for the same letter followed by different letters vary significantly by Tukey’s test (*p* < 0.05). Based on the median TSS (~14.4 °Brix), the population was divided into two groups of high (47 genotypes, 52%) and low (43 genotypes, 48%) TSS content. The asterisk refers to standard commercial colored (red) and bronze (green) cultivars selected as reference genotypes in the current study, including Noble, Majesty, Carlos, and Fry. (**B**) A represented image for the muscadine genotypes exhibited maximal (O43-1-1) and minimal (A18-8-2) TSS content.

The TSS trait showed moderate differences among genotypes with a range of 10.3 °Brix by which muscadine O43-1-1 (18.1 ± 1.2 °Brix), Scarlett (18.1 ± 0.9 °Brix), and the VM O15-16-1 (17.9 ± 1.1 °Brix) genotypes exhibited the highest TSS content with insignificant differences between them. While the muscadine genotype A18-8-2 displayed the lowest TSS content (~7.9 ± 0.5 °Brix). The average TSS among the population was estimated at 14.4 ± 0.2 °Brix. Based on the median TSS (~14.4 °Brix), the population was separated into two main groups that produced high (47 genotypes, 52% of the population) and low TSS content (43 genotypes, 48% of the population). The reference standard commercial cultivar Majesty was listed among muscadine genotypes exhibiting high TSS levels. However, Fry, Noble, and Carlos belonged to the group displaying low TSS content (Figure 1).

#### 3.1.2. Acidity Trait

**Figure 2:**
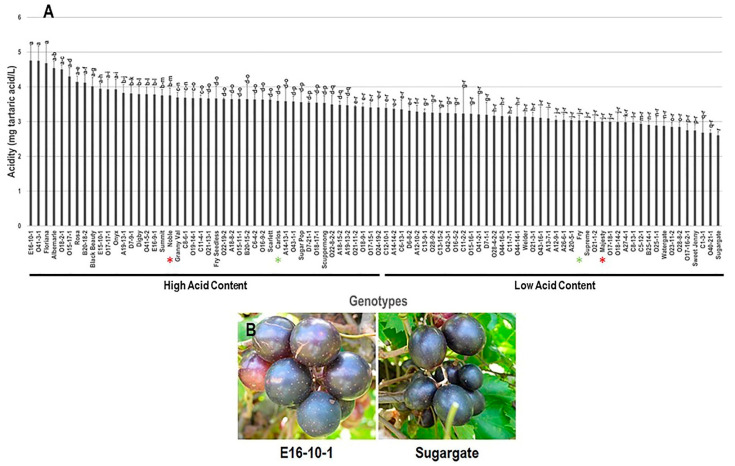
(**A**) Characterization of acidity (Acid) trait among muscadine population (*n* = 90). The bars represent the mean (±SD) acidity levels resulted from three biological and three technical replicates among three years (*n* = 27). The *y*-axis refers to the acidity (mg tartaric acid/L). Means within columns for the same letter followed by different letters differ significantly by Tukey’s test (*p* < 0.05). Based on the median acidity (~3.4 mg tartaric acid/L), the population was divided into two groups of high (46 genotypes, 51.1%) and low (44 genotypes, 48.9%) acid content. Other details are as in Figure 1. (**B**) A represented image for the muscadine genotypes showed maximal (E16-10-1) and minimal Acid (Sugargate).

The acidity trait demonstrated a moderate difference among the population with a range of 2.1 mg tartaric acid/L. The highest acid content was recorded in muscadine genotypes E16-10-1 (4.8 ± 0.3 mg tartaric acid/L), O41-3-1 (4.8 ± 0.4 mg tartaric acid/L), and Floriana (4.7 ± 0.3 mg tartaric acid/L) with no apparent differences among them, whereas muscadine cultivar Sugargate displayed the lowest acid content (2.6 ± 0.2 mg tartaric acid/L)). The average acidity among the population was estimated at 3.4 ± 0.1 mg tartaric acid/L. Based on the median acidity (~3.4 mg tartaric acid/L), the population was divided into two groups of high (46 genotypes, 51.1% of the population) and low (44 genotypes, 48.9% of the population) acid content. Interestingly, the standard commercial wine cultivars, Noble and Carlos, were categorized as members of the muscadine group exhibiting high acid levels. However, the table muscadines Fry and Majesty displayed low acid content (Figure 2).

#### 3.1.3. TSS/Acid (T/A) Ratio Trait

**Figure 3:**
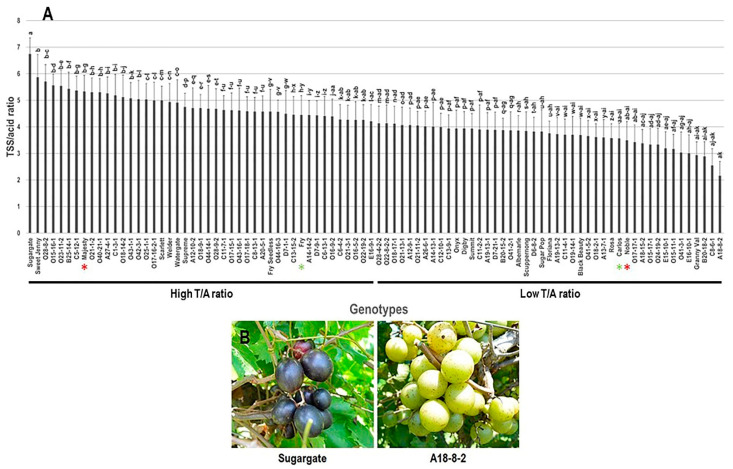
(**A**) Characterization of TSS/Acid ratio (T/A) trait among muscadine population (*n* = 90). The bars represent the mean (±SD) T/A resulted from three biological and three technical replicates among three years (*n* = 27). The *y*-axis refers to the TSS/Acid ratio. Means within columns for the same letter followed by different letters differ significantly by Tukey’s test (*p* < 0.05). Based on the median T/A ratio (~4.2), the population was divided into two equal groups of high and low T/A ratio. Other details are as in Figure 1. (**B**) A represented image for the muscadine genotypes displayed maximal (Sugargate) and minimal T/A ratio (A18-8-2).

The TSS/Acid ratio (T/A ratio) trait displayed a moderate difference among the population with a range of 4.6. The maximum T/A ratio was recorded in Sugargate (6.7 ± 0.6), while the muscadine genotype A18-8-2 displayed the lowest T/A ratio (2.2 ± 0.5). The average T/A ratio among the population was estimated at 4.3 ± 0.1. The median T/A ratio (~3.52) divided the population into two equal groups of high and low T/A ratio. Interestingly, the standard commercial table cultivars were classified as members of the muscadine group, manifesting a high T/A ratio (Figure 3).

#### 3.1.4. pH Trait

**Figure 4:**
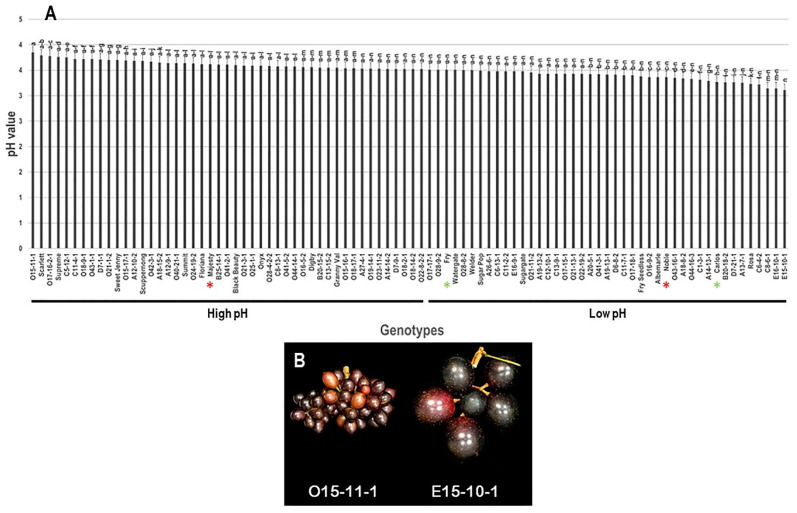
(**A**) Characterization of pH trait among muscadine population (*n* = 90). The bars represent the mean (±SD) pH resulted from three biological and three technical replicates among three years (*n* = 27). The *y*-axis refers to the pH value. Means within columns for the same letter followed by different letters differ significantly by Tukey’s test (*p* < 0.05). Based on the median pH (~3.52), the population was divided into two groups of high manifested (47 genotypes, 52.2%) and low (43 genotypes, 47.8%) pH. Other details are as in Figure 1. (**B**) A represented image for the muscadine genotypes showed maximal (O15-11-1) and minimal pH (E15-10-1).

The pH trait did not confer excessive differences among the population, with a narrow range of 0.74. The highest pH was recorded in the VM genotype O15-11-1 (3.85 ± 0.12), whereas the muscadine genotype E15-10-1 displayed the lowest pH (3.11 ± 0.12). The average pH among the population was estimated at 3.51 ± 0.02. Based on the median pH (~3.52), the population was split into two groups of high (47 genotypes, 52.2% of the population) and low (43 genotypes, 47.8% of the population) pH. According to the previous analysis, the table grape cultivar, Majesty, was placed among the high pH group; however, muscadine cultivars Fry, Noble, and Carlos were located in the low pH class (Figure 4).

#### 3.1.5. Frequency Distribution of Berry Biochemical Traits

**Figure 5:**
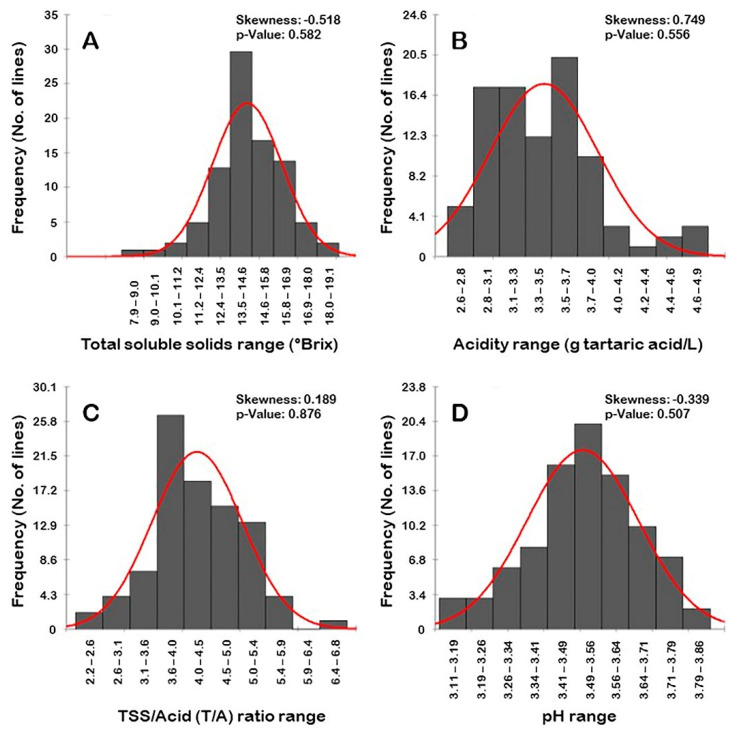
Frequency distribution of the berry biochemical traits, including the total soluble solids (**A**), acidity (**B**), TSS/Acid ratio (**C**), and pH (**D**) in the muscadine population (*n* = 90). The skewness degree and *p*-value of the Kolmogorov–Smirnov normal distribution test are indicated.

Frequency distribution analysis of berry biochemical traits suggested a distinguishable distribution pattern among biochemical parameters (Figure 5). Interestingly, all of the evaluated traits have normal frequency distribution patterns. TSS and pH traits’ distribution behavior was skewed moderately to the left, departing from normality (Figure 5A,D). By contrast, the acidity and T/A characters’ distribution pattern was strongly skewed to the right, departing from normality (Figure 5B,C).

TSS and pH traits typically have a unimodal distribution pattern (Figure 5A,D). This pattern of distribution suggests that both traits were likely regulated in a quantitative manner in the population. The distribution pattern of the T/A ratio trait exhibited a bimodal distribution (Figure 5C). A bimodal distribution usually indicates that two main phenotypes exist in the population, designated as low/moderate T/A ratio (2.2–5.9) that covers almost all the population individuals (89 genotypes or ~98.9% of the population) and high T/A ratio (6.4–6.8) represented by only one genotype (~1.1% of the population). The TSS levels accumulated in muscadine berries are generally lower than in bunch grapes [33]. Accordingly, it is common to find that the T/A ratio among muscadine grapes usually varies between low to moderate. The acidity trait typically has a trimodal distribution in the population. A trimodal distribution typically indicates that three main phenotypes exist in the population (Figure 5B). The three categories were designated as low acid levels (2.6–3.5 g tartaric acid/L; 51 genotypes or ~56.7% of the population), moderate acid levels (3.5–4.2 g tartaric acid/L; 33 genotypes or ~36.7% of the population), and high acid levels (4.2–4.9 g tartaric acid/L; 6 genotypes or ~6.7% of the population).

### 3.2. Color-Related Traits

**Figure 6:**
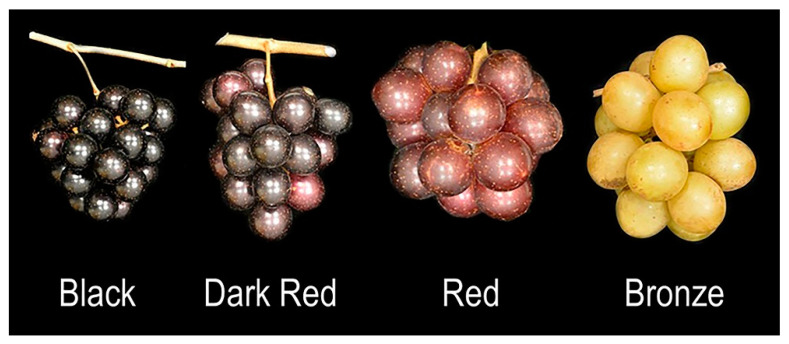
A representative image of muscadine cluster showing different types of berry color among population.

The individuals of the muscadine population were carefully selected to represent the diversity in color berries of muscadine grapes. Based on visual assessment of berry colors, the population was classified into genotypes producing black berries (28 genotypes, 31.1% of the population), dark-red berries (14 genotypes, 15.6% of the population), red berries (6 genotypes, 6.7% of the population), and bronze berries (42 genotypes, 46.7% of the population) (Figure 6).

#### 3.2.1. Total Anthocyanin Content (TAC) Trait

**Figure 7:**
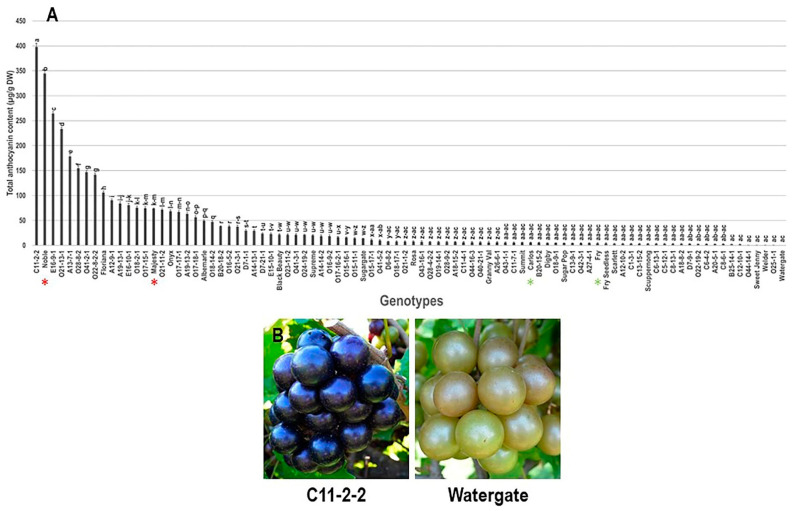
(**A**) Characterization of total anthocyanin content (TAC) trait among muscadine population (*n* = 90). The bars represent the mean (±SD) TAC resulted from three biological and three technical replicates among three years (*n* = 27). The *y*-axis refers to the total anthocyanin content (μg/g DW). Means within columns for the same letter followed by different letters differ significantly by Tukey’s test (*p* < 0.05). Other details are as in Figure 1. (**B**) A represented image for the muscadine genotypes exhibited maximal (C11-2-2) and minimal (Watergate) TAC levels.

Total anthocyanin content (TAC) was measured and calculated as delphinidin-3-diglucoside equivalents, as it has been reported as the most prevalent anthocyanin in muscadine grape berries [27]. As expected, the characterization of the TAC trait revealed broad differences between the individuals within the population with a range of ~398 μg/g DW (Figure 7). The muscadine genotype C11-2-2 had the highest TAC levels (398.1 ± 6.8 μg/g DW). However, several bronze muscadine genotypes displayed the lowest TAC levels with no significant differences among them. This comprises the muscadine genotypes Watergate (0.2 ± 0.1 μg/g DW), O25-1-1 (0.3 ± 0.1 μg/g DW), Welder (0.3 ± 0.1 μg/g DW), Sweet Jenny (0.3 ± 0.1 μg/g DW), and O44-14-1 (0.3 ± 0.1 μg/g DW). The average TAC value among the population was estimated at 39.1 ± 7.5 μg/g DW. The median TAC observed was ~7.6 μg/g DW. It is important to highlight that the assessment of the TAC trait was not efficiently able to distinguish between colored and non-colored muscadine berries since there are several dark red berry genotypes (i.e., C11-7-1 and O19-14-1) demonstrated TAC levels lower than those detected in bronze genotypes (i.e., A18-15-2 and O18-17-1). Despite that, the two-colored cultivars, Noble and Majesty, and the two non-colored ones, Carlos and Fry, were grouped in a color-dependent manner.

#### 3.2.2. Color Luminosity Index (L*) Trait

**Figure 8:**
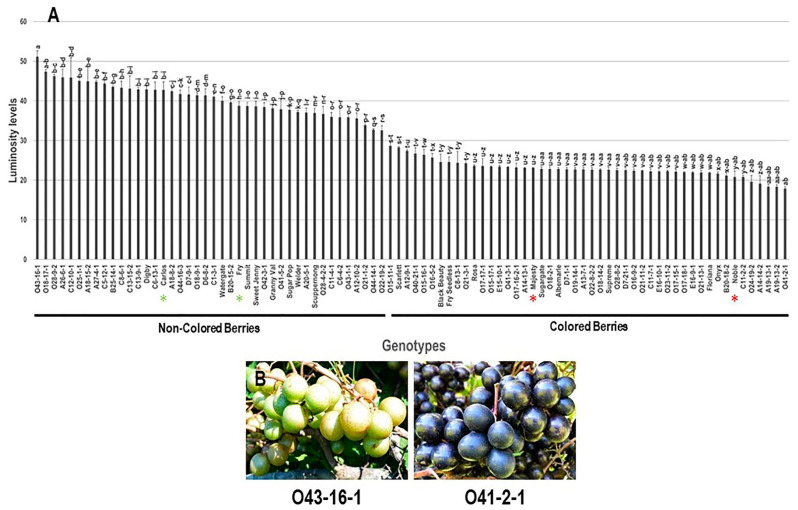
(**A**) Characterization of luminosity index (L*) trait among muscadine population (*n* = 90). The bars represent the mean (±SD) L* value resulted from three biological and three technical replicates among three years (*n* = 27). The *y*-axis refers to the color lightness. Means within columns for the same letter followed by different letters differ significantly by Tukey’s test (*p* < 0.05). Based on the estimated median luminosity (~27.0), the population was divided into two groups of non-colored/high L* index (42 genotypes, 46.7%) and colored/low L* index (48 genotypes, 53.3%). Other details are as in Figure 1. (**B**) A represented image for the muscadine genotypes displayed maximal (O43-16-1) and minimal (O41-2-1) L* values.

Analysis of color lightness index (L*) trait among the population stated that the lower L* values were associated with colored berries, while the higher L* values were concomitant with non-colored berries. The L* values visibly varied among the population and displayed a wide range of ~33.2. Bronze muscadine berry genotype O43-16-1 had the highest L* value of 51.1 ± 1.5, while the black berry genotype O41-2-1 displayed the lowest L* value, 17.9 ± 0.5. The population had an average of 31.1 ± 1.0. Based on the estimated median luminosity (~27.0), the population was classified into two main groups of non-colored/high L* index (42 genotypes, 46.7% of the population) and colored/low L* index (48 genotypes, 53.3% of the population), which perfectly match with the visual assessment of berry color (Figure 8). Accordingly, the two muscadine cultivars, Noble and Majesty, were placed among the colored group; however, the muscadine cultivars, Fry and Carlos, were located in the non-colored class.

#### 3.2.3. Hue Angle (*h*°) Trait

**Figure 9:**
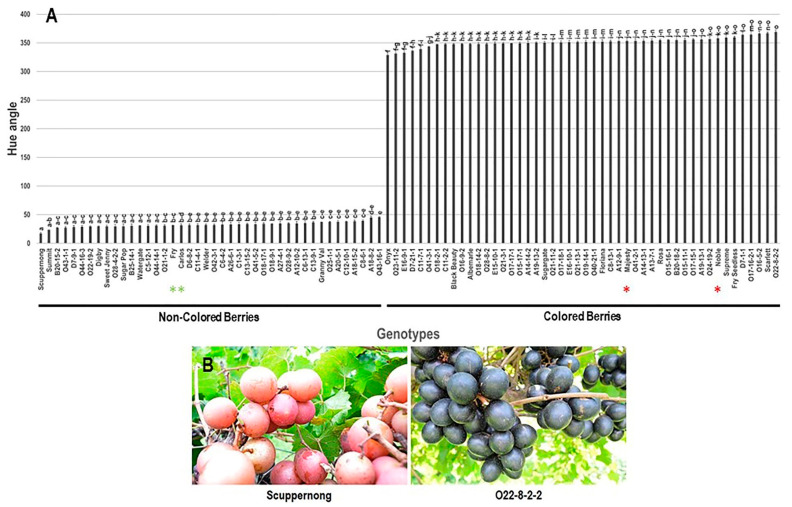
(**A**) Characterization of hue angle (*h*°) trait among muscadine population (*n* = 90). The bars represent the mean (±SD) *h*° value resulted from three biological and three technical replicates among three years (*n* = 27). The *y*-axis refers to the hue angle. Means within columns for the same letter followed by different letters differ significantly by Tukey’s test (*p* < 0.05). Based on *h*° characterization, the population was divided into two groups of low (non-colored, 42 genotypes, 46.7%) and high (colored, 48 genotypes, 53.3%) *h*° values. Other details are as in Figure 1. (**B**) A represented image for the muscadine genotypes demonstrated minimal (Scuppernong) and maximal (O22-8-2-2) *h*° values.

Contrary to L*, analysis of color hue angle (*h*°) trait among the population identified that the lower *h*° values were associated with non-colored berries. In comparison, the higher *h*° values were correlated with colored berries. The population demonstrated a wide *h*° value range of ~352.1, with a mean of 202.6 ± 16.9 (Figure 9). The bronze Scuppernong muscadine cultivar presented the minimum *h*° recorded with a value estimated at 17.0 ± 1.7. On the other side, the black muscadine genotype O22-8-2-2 displayed the maximum *h*° value estimated at 369.1 ± 3.3. Based on the median *h*° (~334.3), the population was classified into two groups of low *h*° values, and this includes all the non-colored, bronze genotypes (42 genotypes, 46.7% of the population), and high *h*° values that comprises all the colored, black/dark red/red genotypes (48 genotypes, 53.3% of the population), which ideally corresponded with the visual evaluation of berry color. Accordingly, the four muscadine cultivars were allocated in their relevant groups.

#### 3.2.4. Chroma Index (C*) Trait

**Figure 10:**
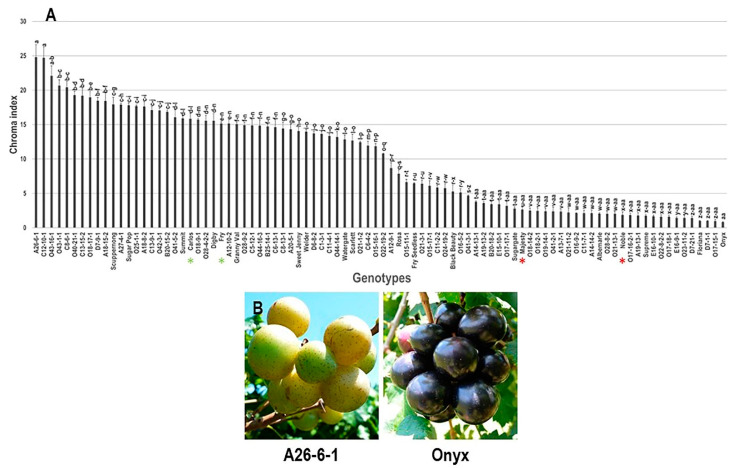
(**A**) Characterization of chroma index (C*) trait among muscadine population (*n* = 90). The bars represent the mean (±SD) C* value resulted from three biological and three technical replicates among three years (*n* = 27). The *y*-axis refers to the chroma index. The *x*-axis refers to muscadine genotypes. Means within columns for the same letter followed by different letters differ significantly by Tukey’s test (*p* < 0.05). Other details are as in Figure 1. (**B**) A represented image for the muscadine genotypes showed maximal (A26-6-1) and minimal (Onyx) C* values.

Analysis of berry C* index trait revealed a wide chroma range of 24 among the population (Figure 10). The two bronze muscadine genotypes, A26-6-1 and C12-10-1, recorded the highest C* index estimated at 24.8 ± 1.8 and 24.7 ± 1.6, respectively, without significant difference between them. The lowest C* value was detected in the black berry muscadine cultivar Onyx with a C* index estimated by 0.8 ± 0.1. The mean C* value among the population was estimated at 9.8 ± 0.7; however, the median chroma was calculated at ~11.3. As in the TAC trait, the assessment of C* could not distinguish between colored and non-colored berries since some red berry genotypes (i.e., O15-16-1) exhibited similar C* values to the bronze genotypes (i.e., O22-19-2).

#### 3.2.5. Frequency Distribution of Color-Related Traits

**Figure 11:**
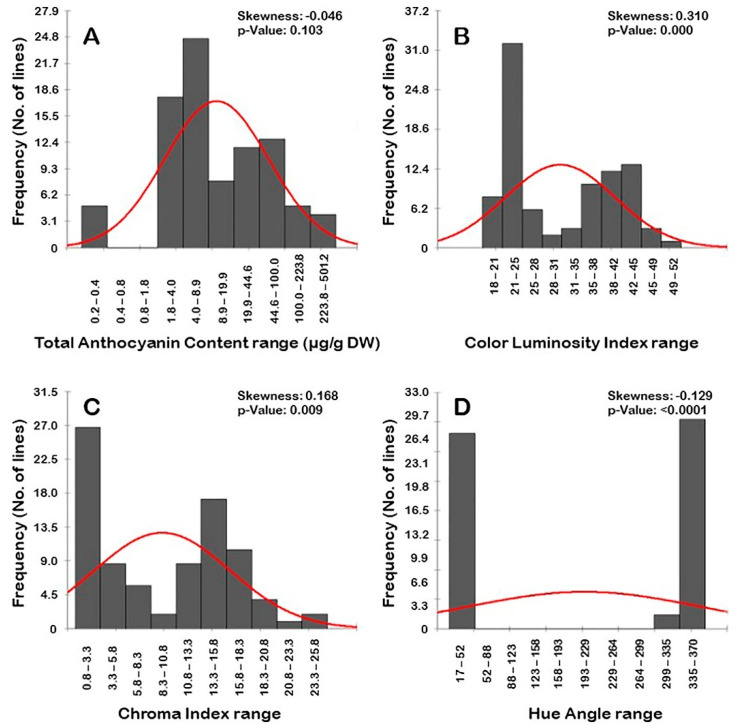
Frequency distribution of the berry color traits, including total anthocyanin content (**A**), luminosity index (**B**), chroma index (**C**), and hue angle (**D**) in the muscadine population (*n* = 90). The skewness degree and *p*-Value of the Kolmogorov–Smirnov normal distribution test are indicated.

Frequency distribution analysis of berry color-related traits suggested a distinct distribution pattern among traits (Figure 11). The evaluated traits did not follow a normal frequency distribution pattern, excluding the TAC parameter. The distribution behavior for TAC and *h*° traits were skewed slightly to the left, departing from normality (Figure 11A,D). By contrast, the distribution pattern for L* and C* means were skewed to the right, departing from normality (Figure 11B,C).

The TAC, L*, and C* traits typically have a trimodal distribution in the population. A trimodal distribution pattern suggested three main phenotypes exist in the population (Figure 11A–C). In the case of TAC, the three categories were designated as low TAC levels (0.2–0.4 μg/g DW; 5 genotypes, or ~5.6% of the population), moderate TAC levels (1.8–19.9 μg/g DW; 51 genotypes, or ~56.7% of the population), and high TAC levels (19.9–501.2 μg/g DW; 34 genotypes, or ~37.8% of the population). The L* trait was divided into three groups based on luminosity level, designated as low lightness (18–32; 48 genotypes, or ~53.3% of the population), moderate lightness (32–42 g; 25 genotypes, or ~27.8% of the population), and high lightness (42–52 g; 17 genotypes, or ~18.9% of the population). It is important to highlight that the first group covers all colored genotypes (black/dark red/red). The second group includes all bronze genotypes. The third group comprises all green/yellowish genotypes. Finally, for the C* trait, the three categories were manifested as low C* levels (0.8–10.8; 45 genotypes or ~50% of the population), moderate C* levels (10.8–23.3; 43 genotypes or ~47.8% of the population), and high C* levels (23.3–25.8; 2genotypes or ~2.2% of the population).

The distribution pattern of the *h*° trait exhibited a bimodal distribution (Figure 11D). A bimodal distribution usually indicates that two main phenotypes exist in the population, designated as low *h*° values that cover all non-colored genotypes (17–52; 42 genotypes or ~46.7% of the population) and high *h*° values that include all colored genotypes (299–370; 48 genotypes or ~53.3% of the population). The pattern was evident, suggesting that the trait in muscadine is controlled by one to two genes.

#### 3.2.6. Classification of Muscadine Genotypes Based on the Evaluated Traits

**Figure 12:**
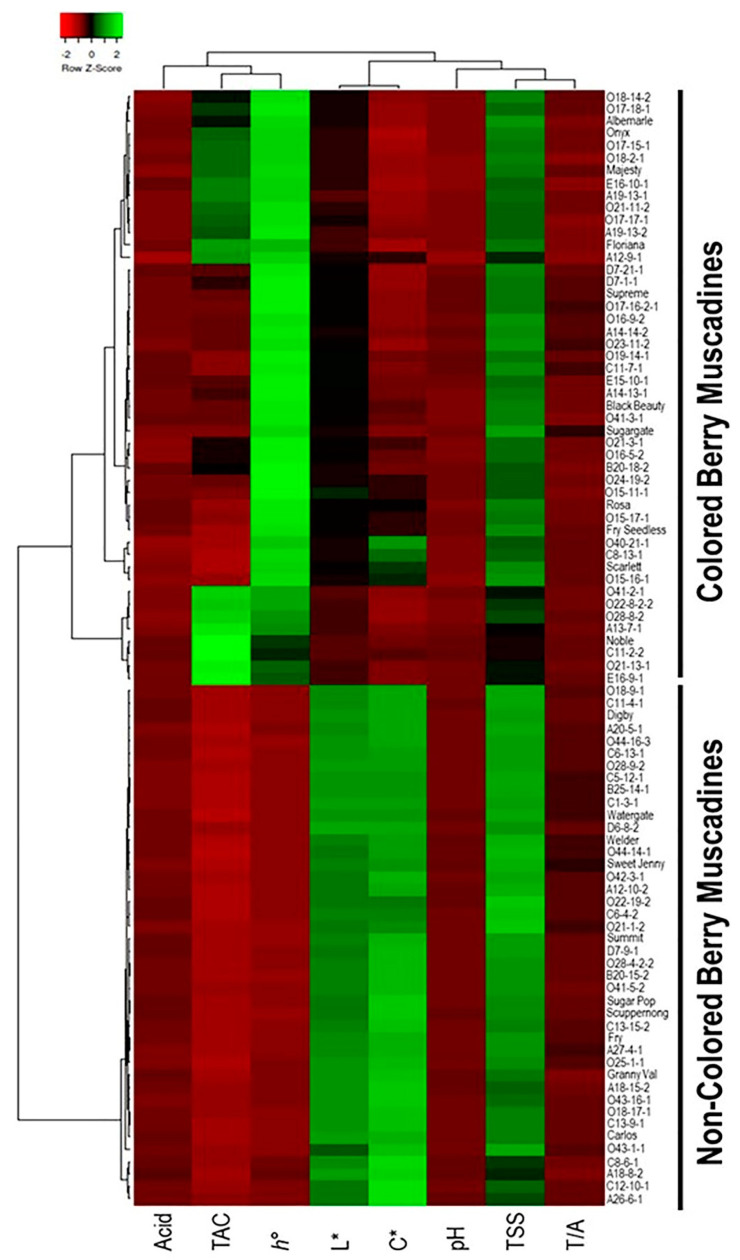
Hierarchical clustering of the different evaluated traits of muscadine population (*n* = 90). Data related to TSS, Acid, T/A ratio, pH, TAC, L*, *h*°, and C* are presented as an average of three biological and three technical replicates, among three years (*n* = 27). The log2-transformed values of each character are represented by colors. Green and red boxes indicate higher and lower values, respectively. The color change is proportional to the two extremes (see the color scale at the top of the figure).

In order to classify the muscadine population based on the evaluated biochemical and color-related traits, a hierarchical cluster map was constructed (Figure 12). Despite the variation among individuals for the different evaluated traits, the hierarchical clustering divided the population into two main groups of colored and non-colored grapes based on luminosity index and hue angle, suggesting the dominance of these two characters among the studied population. The clade of non-colored muscadines was slightly branched after that, suggesting uniform levels of the remaining traits. On the other hand, the colored clade was extensively separated into several sub-clades, mainly due to differences in C*, TAC, and TSS levels.

#### 3.2.7. Dissimilarity Matrix Analysis among the Population

**Figure 13:**
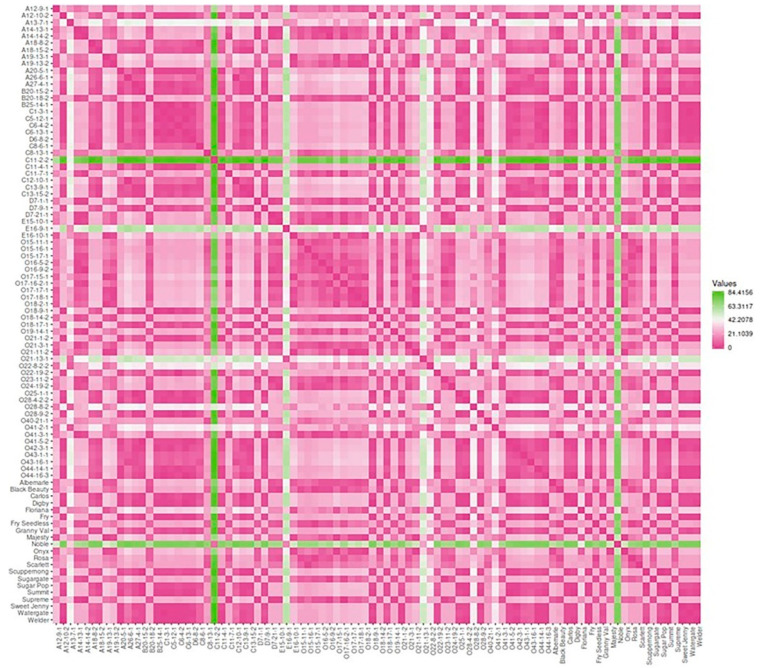
Dissimilarity matrix showing the distances among the genotypes. The gradient of color indicates the distance between genotypes; the green color denotes the highest dissimilarity, and the pink color means the lowest genetic distance. In addition, the pink color represents the diagonal.

The 90 genotypes analyzed in this study showed a high level of diversity with little population structure. The dissimilatory matrix revealed an overall low level of relatedness with very few pairs of closely related genotypes within the population (Figure 13). Despite the distinct background of muscadines and VMs, the VM genotypes were not distinguishable from muscadines. Interestingly, some muscadine genotypes were noticeably divergent from the population, including C11-2-2, E16-9-1, O21-13-1, and Noble. Data analysis revealed the enhanced color characteristics of the highlighted genotypes.

#### 3.2.8. Principal Component Analysis of Different Evaluated Traits

**Figure 14:**
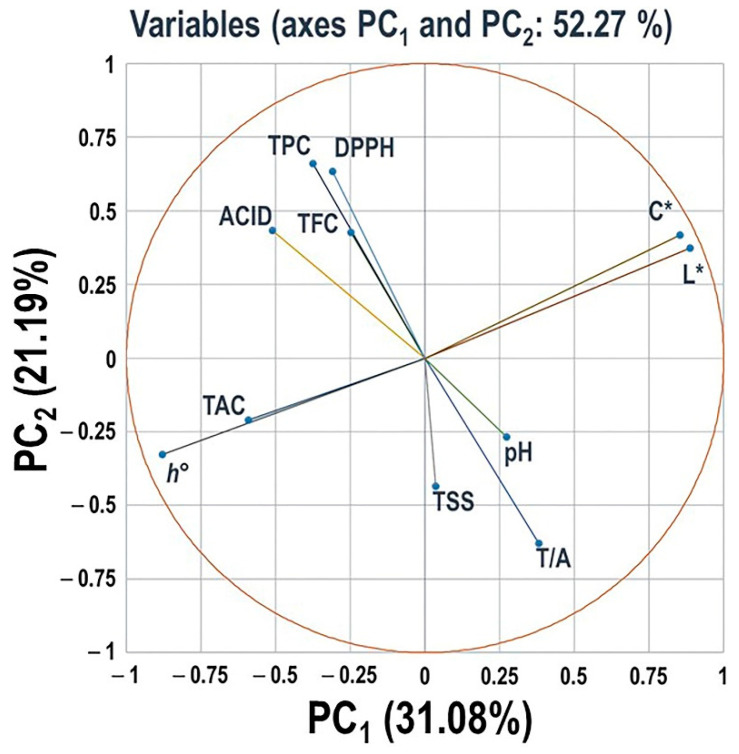
Principal Component Analysis (PCA) scatter plots of different berry-related traits, including total soluble solids (TSS), acidity (Acid), TSS/Acid ratio (T/A), pH, total anthocyanin content (TAC), luminosity index (L*), hue angle (*h*°), and chroma index (C*). Other previously characterized traits for the same muscadine population, including total phenolic content (TPC), total flavonoid content (TFC), and DPPH antioxidant activity, were added to the analysis. The scatter was generated using the average of three biological and three technical replicates, among three years (*n* = 27). According to the PCA model, 31.08% and 21.19% of the variance were explained by the PC1 and the PC2 principal components, respectively.

Principle components analysis (PCA) was performed using the data from the assessment of 90 muscadine genotypes to identify different groups of berry-related traits coordinating biochemical and color characteristics (Figure 14). However, to get a better perception of muscadine berry properties, we have included previously assessed nutraceutical data related to total phenolic content (TPC), total flavonoid content (TFC), and DPPH antioxidant activity by which the same muscadine individuals in the current study were used [32].

Previous results suggested that the TPC trait varied widely among the population with a range of ~137.8 mg GAE/g DW. The muscadine genotypes O43-1-1, O43-16-1, and O41-3-1 had the highest TPC levels (144–152.5 mg GAE/g DW); however, the O24-19-2 displayed the lowest detectable TPC (14.7 ± 1.3 mg GAE/g DW). Three genotypes, including the bronze muscadine O44-16-3 (97.8 ± 8.1 mg QE/g DW), the dark red VM O15-17-1 (91.1 ± 8.4 mg QE/g DW), and the red VM O15-16-1 (85.6 ± 7.0 mg QE/g DW), displayed the highest TFC levels with no significant differences among them. Whereas the muscadine C11-7-1 showed the lowest TFC levels (14.9 ± 1.1 mg QE/g DW). The black VM genotype O16-9-2 had the highest DPPH activity (56.9% ± 5.2); however, the O24-19-2 displayed the lowest DPPH levels (7.1% ± 0.5) [32].

For the PCA, the variances explained by the first two components were 31.08 and 21.19%, respectively, with 52.27% cumulative eigenvalues of data variance. Eigenvalues of the third and fourth PCs were negligible (48.38% and 39.99%), and thereby they are not discussed further. The PC1 showed a strong positive correlation with chroma index (C*) and luminosity index (L*), as well as a definite negative correlation with total anthocyanin content (TAC) and hue angle (*h*°). The PC2 displayed a strong positive correlation with the variables of acidity, TPC, TFC, and DPPH antioxidant activity. However, it exhibited a clear negative correlation with the TSS, TSS/Acid (T/A) ratio, and pH.

## 4. Discussion

Information about juice attributes and color of muscadine grapes can afford valuable insights for product marketability, consumer preference, and the food processing industry. Furthermore, the characterization of biochemical properties of muscadine grapes should facilitate their classification in terms of flavor and taste for the viticulture industry and grape breeders. In the current study, the biochemical-related traits, including TSS, acidity, T/A ratio, and juice pH, were determined. The flavor composition has been defined as a complex attribute of quality in which the assortment of sugars and acids plays a primary role [34]. Volatile esters, sugar, and organic acids, many of which are intermediates in metabolic processes, play significant roles in fruit growth, maturation, ripening, and softening. In grapes, the sugar and organic acids’ levels greatly influence the taste and flavor of the berry, the quality of a processed product, and, consequently, determine its ultimate market value [10,35]. On the other hand, sugar and organic acids contribute to the juice’s sensory quality because they ensure the balance between sweet and acid flavors, which confers appreciated palatability [36].

TSS, acidity, and T/A ratio are broadly accepted parameters commonly used as grape maturity indicators [37]. At harvest, the total sugars account for more than 90% of the TSS [10]. The sugars in ripe berries are present at high concentrations in the flesh [38,39]. It has been reported that berry density and size are related aspects to the physicochemical properties of grapes [40]. Earlier studies suggested the reduction of sugar concentration along with increasing berry size [41,42]. Roby et al. [43] suggested the positive correlation between berry size and anthocyanin content. In this study, TSS content showed a moderate range among the muscadine population (7.9–18.1 °Brix). Glucose and fructose are the major sugars accumulated in bunch grape berries (*Vitis*–based grapes) due to sucrose hydrolyzing and metabolism [41,44]. The enzymes responsible for sugar metabolism in bunch grape berry are sucrose phosphate synthase, sucrose synthase, and invertase [45]. Sucrose is cut into glucose and fructose in the berry by invertase or sucrose synthase [16]. It seems that the variation in the TSS content between muscadine genotypes is due to differences in aforesaid enzyme activity.

T/A ratio is a sign of grape ripeness, as the concentration of sugars and organic acids under similar conditions varies from one year to another [46]. Organic acids are the most notable contributors to the ultimate berry taste [47,48]. The primary organic acids of grape juice are tartaric, malic, and citric acids, but small quantities of other acids are present. Out of these three acids, tartaric and malic acids account for over 90% of the total acid constituents of the juice [49]. The organic acids content and the balance between sugar and acid (T/A ratio) are the largest determining factors for grape berry quality at harvest, particularly those used for wine production. In this study, the acidity trait displayed modest but significant differences among the population (2.6–4.8 g tartaric acid/L). In general, the organic acid content in grapes is influenced by several factors such as grape variety, degree of ripeness, growing region, level of insolation, climatic conditions, and storage conditions or duration [12,13,50,51]. These components are essential signals in determining the ripeness of the grape and the flavor of its derivatives [48]. The T/A ratio trait also displayed significant differences among the population (2.2–6.8). The varied T/A ratio among the muscadine population was different. The cause of the high T/A ratio could be due to high TSS coincided with low acid contents or resulted from balanced low TSS and reduced acid accumulations. However, the low T/A ratio was because of low TSS and high acid contents. PCA analysis showed that the higher T/A ratio is usually associated with higher TSS and pH variables. The grapes juice pH is also a critical factor for flavor and resistance to spoilage during postharvest storage and downstream processing [52]. Grape juice is acidic, with a pH that generally has a range of 3.2 to 4.0. In this study, the juice pH of the muscadine genotypes did not show a significant difference among the population (3.11–3.85).

Color is considered the basis for the quality assessment due to its aesthetic role and nutrition value [53]. Breeding cultivars with new skin colors may open up new markets for muscadines [54]. The skin color of grape berries is a critical trait that greatly influences the end-use of the fruit. In both wine and table grapes, fruit color is a decisive breeding target; both noir (“red,” “blue,” or “black”) and non-noir (“green” or “white”) grapes can be desirable, depending on the intended use [55]. In table grapes, berry color has been shown to influence consumer preference [56], while wine grape color influences the color of the final wine produce. In the current study, the color of grape berries was evaluated using different factors, including total anthocyanin content (TAC), luminosity index (L*), hue angle (*h*°), and chroma index (C*). All color-related traits visibly varied among the population. The lower and higher L* values were associated with colored berries and non-colored berries, respectively. In color theory, according to Conway [57,58], *h*° refers to a pure color. Contrary to the color luminosity trait, the higher hue angle values were associated with colored berries, while lower *h*° values were allied to non-colored berries. Chroma index refers to the degree of vividness or intensity of a color. In another meaning, how the color is pure or saturated compared to its representative on the color wheel. Like the luminosity index, the lower C* values were associated with colored berries, while higher C* values were linked to non-colored.

The total anthocyanin content and the proportions with color determine the final coloration of the grape skin [10]. It has been reported that the anthocyanins were unevenly distributed in different parts of muscadine grapes. However, anthocyanins were mainly accumulated in the skin [5]. Total anthocyanin content (TAC) and relative amounts of individual anthocyanins are significantly correlated with the CIELAB coordinates of L*, a*, and b* [59]. Through evaluating 32 red grape varieties, Carreno et al. [60] determined that amounts of anthocyanins in grape skin varied between 6.3–201 mg/100 g. In addition, total anthocyanin in 20 black and bronze muscadine cultivars varied among cultivars by which black cultivars had the highest total anthocyanin [61,62]. In this study, the total anthocyanin content (TAC) trait revealed vast differences within the population (0.2–398.1 µg/g DW), where bronze muscadine genotypes displayed substantially the lowest TAC levels. It has been reported that accountable for blue, red, and purple colors, five anthocyanins were detected in *Vitis vinifera* species, including malvidin, petunidin, peonidin, cyanidin, and delphinidin [63]. Identification of anthocyanins in muscadine grapes showed that approximately 90% of the total anthocyanins were 3,5-diglucoside of delphinidin, cyanidin, and petunidin; the remaining 10% were 3,5-diglucoside of peonidin and malvidin. The dominant allele involved in the production of diglucosidic anthocyanins is not present in *V. vinifera*, resulting in the sole production of 3-*O*-monoglucosides [64]. In contrast, other grape species, including muscadine, can form 3,5-*O*-diglucosidic anthocyanins. The 3,5-diglucosides may be more resistant to thermal degradation than the monoglucosides, but they have a diminished ability to form polymeric pigments, making them more prone to oxidation and browning [22,65,66]. Ballinger et al. [27] examined the anthocyanin profile of several *M. rotundifolia* clones and found that delphinidin was the predominant anthocyanin. Despite the predominance of delphinidin, delphinidin content was not associated with visual color ratings of the berries and wines. Wines with good red color were strongly associated with high contents of malvidin and, to a lesser extent, with petunidin. Population data analysis suggested that only L* and *h*° traits could distinguish between colored and non-colored muscadine berries population. However, the TAC and C* traits could not fully distinguish between colored and non-colored muscadine berries. Several red berry genotypes displayed lower TAC levels and higher C* values than those in bronze genotypes. This suggested the contribution of the level of total anthocyanin and the type of dominant anthocyanin accumulated. Accordingly, HPLC analysis with a larger number of muscadine individuals is needed to confirm this hypothesis. The hierarchical cluster map confirmed the contribution of L* and *h*° via separating the population into two large groups of colored and non-colored grapes based on these two variables.

PCA analysis, including nutraceutical parameters of muscadine grapes, resulted in two unexpected correlations. It highlighted the significant contribution of acidity to enhanced TPC, TFC, and, consequently, antioxidant activity. The fruit possesses abundant organic acids with different and complex compositions that can influence the polyphenol structure of the samples [67]. Among all the detected organic acids in fruits, the tartaric acid and malic acid (the most abundant acids in the muscadine grape) should be responsible for enhanced antioxidant activity [68]. One of the most pronounced results from the PCA study was the minor contribution of TAC to TPC, TFC, and DPPH. Anthocyanins are colored, water-soluble pigments belonging to the phenolic group. In several fruit species, the contribution of anthocyanin to the antioxidant activity and nutraceutical value, including grapes, is evident [69]. It is tempting to speculate that the nature of anthocyanins accumulated in muscadine grapes, as diglucoside anthocyanins, might influence their bioactivities. Antioxidant assays using pure anthocyanins extracted from muscadine grapes are needed to confirm this hypothesis. Overall, a better understanding of berry color, led by improved phenotyping techniques, will help grape breeders target desirable color profiles and breed for them more efficiently.

## 5. Conclusions

The current study is the first comparative investigation on juice attributes and skin color of 90 muscadine grape genotypes. Biochemical juice attributes and color of muscadine grapes showed significant differences among the population. In addition, the TAC level is not enough to determine the quality of berry color. Other components, such as L*, *h*°, and C*, should support the evaluation of berry color. The muscadine population in this germplasm collection had significant diversity for the breeding program, leading to more potential in the nutraceutical arena. Overall, the comparative study on muscadine grape genotypes can better improve our understanding of berry color and assist grape breeders in selecting the desired color profiles. Based on overall performance, the muscadine genotypes B20-18-2 and C11-2-2 were selected as an advanced selection to be monitored for another season, suitable for red wine production.

## Data Availability

The data presented in this study are available to anyone upon request.

## Supplementary Materials

The following are available online at https://www.mdpi.com/article/10.3390/foods10051101/s1, Table S1: List of used genotypes and parental pedigree.
