# Supplementary material for: Evaluation of Biochemical Juice Attributes and Color-Related Traits in Muscadine Grape Population
Source: Foods. 2021 May 16;10(5):1101. doi: 10.3390/foods10051101 (PMC8156615; doi:10.3390/foods10051101)
Supplement: Supplementary file 1 [file foods-10-01101-s001.zip › foods-1207097-supplementary.pdf]

| Sample | Genotype | Species | Parents of Individual | | Optimal Harvest Date |
|---|---|---|---|---|---|
| | | | Female | Male | |
| | | | | | |
| 1 | A12-9-1 | Muscadine hybrid | Hunisia | Alachua | September; FRW |
| 2 | A12-10-2 | Muscadine hybrid | Hunisia | Alachua | September; SEW |
| 3 | A13-7-1 | Muscadine hybrid | UNK | UNK | September; FRW |
| 4 | A14-13-1 | M. rotundifolia | Onyx | UNK | July; FOW |
| 5 | A14-14-2 | M. rotundifolia | Onyx | UNK | August; FOW |
| 6 | A18-8-2 | M. rotundifolia | UNK | UNK | August; FOW |
| 7 | A18-15-2 | M. rotundifolia | Majesty | Ison | August; FOW |
| 8 | A19-13-1 | M. rotundifolia | Onyx | UNK | August; FOW |
| 9 | A19-13-2 | M. rotundifolia | Onyx | UNK | August; THW |
| 10 | A20-5-1 | M. rotundifolia | Onyx | UNK | August; FOW |
| 11 | A26-6-1 | M. rotundifolia | O26-5-8 (Supreme X Triumph) | UNK | August; THW |
| 12 | A27-4-1 | M. rotundifolia | Sweet Jenny | Tara | August; THW |
| 13 | B20-15-2 | M. rotundifolia | Darlene | UNK | August; THW |
| 14 | B20-18-2 | M. rotundifolia | UNK | UNK | August; THW |
| 15 | B25-14-1 | M. rotundifolia | Pam | UNK | August; THW |
| 16 | C1-3-1 | M. rotundifolia | Pam | UNK | August; SEW |
| 17 | C5-12-1 | M. rotundifolia | Fry | Tara | August; THW |
| 18 | C6-4-2 | M. rotundifolia | UNK | UNK | August; SEW |
| 19 | C6-13-1 | M. rotundifolia | Early Fry | UNK | August; FRW |
| 20 | D6-8-2 | M. rotundifolia | O25-15-7 (Supreme X UNK) | Triumph | August; FOW |
| 21 | C8-6-1 | M. rotundifolia | Supreme | Tara | September; FRW |
| 22 | C8-13-1 | M. rotundifolia | Supreme | UNK | August; FOW |
| 23 | C11-2-2 | M. rotundifolia | Fry | UNK | August; FOW |
| 24 | C11-4-1 | M. rotundifolia | UNK | UNK | August; SEW |
| 25 | C11-7-1 | M. rotundifolia | Onyx | UNK | August; FOW |
| 26 | C12-10-1 | M. rotundifolia | Sweet Jenny | Tara | August; THW |
| 27 | C13-9-1 | M. rotundifolia | Supreme | Triumph | September; FRW |
| 28 | C13-15-2 | M. rotundifolia | O21-2-4 (Pam X UNK) | UNK | August; THW |
| 29 | D7-1-1 | M. rotundifolia | O25-15-7 (Supreme X UNK) | Triumph | August; THW |
| 30 | D7-9-1 | M. rotundifolia | O25-15-7 (Supreme X UNK) | Triumph | August; THW |
| 31 | D7-21-1 | M. rotundifolia | Ison | Tara | August; FOW |
| 32 | E15-10-1 | M. rotundifolia | Pam | Nesbitt | August; SEW |
| 33 | E16-9-1 | M. rotundifolia | Pam | Noble | August; SEW |
| 34 | E16-10-1 | M. rotundifolia | Pam | Noble | September; SEW |
| 35 | O15-11-1 | Muscadine hybrid | A11-1-4 | A11-1-4 | September; THW |
| 36 | O15-16-1 | Muscadine hybrid | A11-1-4 | A11-1-4 | September; THW |
| 37 | O15-17-1 | M. rotundifolia | Darlene | UNK | September; THW |
| 38 | O16-5-2 | Muscadine hybrid | A11-1-4 | A11-1-4 | September; THW |
| 39 | O16-9-2 | Muscadine hybrid | A11-1-4 | A11-1-4 | August; THW |
| 40 | O17-15-1 | M. rotundifolia | Majesty | Ison | August; FOW |
| 41 | O17-16-2-1 | M. rotundifolia | Majesty | Ison | August; FOW |
| 42 | O17-17-1 | M. rotundifolia | Majesty | Ison | August; FRW |
| 43 | O17-18-1 | M. rotundifolia | Majesty | Ison | August; FRW |
| 44 | O18-2-1 | M. rotundifolia | O23-1-5 (Pam X UNK) | UNK | August; FOW |
| 45 | O18-9-1 | M. rotundifolia | O23-1-5 (Pam X UNK) | UNK | August; THW |
| 46 | O18-14-2 | M. rotundifolia | O23-1-5 (Pam X UNK) | UNK | August; FOW |

| | | | | | |
|---|---|---|---|---|---|
| 47 | O18-17-1 | M. rotundifolia | Fry | Tara | August; SEW |
| 48 | O19-14-1 | M. rotundifolia | Ison | Triumph | August; FOW |
| 49 | O21-1-2 | M. rotundifolia | Supreme | Triumph | August; SEW |
| 50 | O21-3-1 | M. rotundifolia | Supreme | Triumph | August; THW |
| 51 | O21-11-2 | Muscadine hybrid | Triumph | A2-14-2 | August; THW |
| 52 | O21-13-1 | Muscadine hybrid | Triumph | A2-14-2 | August; FOW |
| 53 | O22-8-2-2 | M. rotundifolia | Majesty | Ison | August; FOW |
| 54 | O22-19-2 | M. rotundifolia | Majesty | UNK | August; SEW |
| 55 | O23-11-2 | M. rotundifolia | Majesty | UNK | August; FOW |
| 56 | O24-19-2 | M. rotundifolia | Majesty | Ison | August; SEW |
| 57 | O25-1-1 | M. rotundifolia | Majesty | UNK | August; FOW |
| 58 | O28-4-2-2 | M. rotundifolia | Supreme | Tara | August; SEW |
| 59 | O28-8-2 | M. rotundifolia | Supreme | Tara | August; SEW |
| 60 | O28-9-2 | M. rotundifolia | Supreme | Tara | August; FOW |
| 61 | O40-21-1 | M. rotundifolia | Supreme | UNK | August; FOW |
| 62 | O41-2-1 | M. rotundifolia | Black Beauty | UNK | August; SEW |
| 63 | O41-3-1 | M. rotundifolia | Black Beauty | UNK | August; FOW |
| 64 | O41-5-2 | M. rotundifolia | Darlene | UNK | August; SEW |
| 65 | O42-3-1 | M. rotundifolia | Early Fry | UNK | September; SEW |
| 66 | O43-1-1 | M. rotundifolia | Pam | UNK | August; FOW |
| 67 | O43-16-1 | M. rotundifolia | Late Fry | UNK | August; FOW |
| 68 | O44-14-1 | M. rotundifolia | Pam | Nesbitt | August; FOW |
| 69 | O44-16-3 | M. rotundifolia | Pam | Southern Home | August; SEW |
| 70 | Albemarle | M. rotundifolia | Topsail | Burgaw | August; THW |
| 71 | Black Beauty | M. rotundifolia | Fry | 12-12-1 | August; FOW |
| 72 | Carlos | M. rotundifolia | Howard | NC11-173 (Topsail X Tarheel) | August; THW |
| 73 | Digby | M. rotundifolia | UNK | UNK | August; THW |
| 74 | Floriana | M. rotundifolia | Supreme | Pineapple | August; THW |
| 75 | Fry | M. rotundifolia | Ga.S.19-13 | USDA19-11 | August; FOW |
| 76 | Fry Seedless | M. rotundifolia | Farrer | Redgate | August; SEW |
| 77 | Granny Val | M. rotundifolia | Fry | Carlos | September; FRW |
| 78 | Majesty | M. rotundifolia | Black Fry | Dixieland | August; THW |
| 79 | Noble | M. rotundifolia | Thomas | Tarheel | August; THW |
| 80 | Onyx | M. rotundifolia | Supreme | Ison | August; THW |
| 81 | Rosa | M. rotundifolia | UNK | UNK | September; FRW |
| 82 | Scarlett | M. rotundifolia | Summit | Triumph | August; FOW |
| 83 | Scuppernong | M. rotundifolia | UNK | UNK | August; FOW |
| 84 | Sugargate | M. rotundifolia | Fry | Ga.S.29-49 | July; FOW |
| 85 | Sugar Pop | M. rotundifolia | UNK | UNK | August; FOW |
| 86 | Summit | M. rotundifolia | Fry | Ga.S.29-49 | August; FOW |
| 87 | Supreme | M. rotundifolia | Black Fry | Dixieland | August; FOW |
| 88 | Sweet Jenny | M. rotundifolia | 11-2-2 | 12-12-1 | August; THW |
| 89 | Watergate | M. rotundifolia | UNK | UNK | August; THW |
| 90 | Welder | M. rotundifolia | Dearing | UNK | August; THW |
| **UNK** means unknown parent.<br>**FRW:** first week; **SEW:** second week; **THW:** third week; **FOW:** fourth week. | | | | | |
